# A Warning against Antifebrin

**Published:** 1898-12

**Authors:** 


### A WARNING AGAINST ANTIFEBRIN.

An inquest was recently held in London on the body of a young man who died from taking powders containing an indeterminate quantity of antifebrin. The powders were taken for the relief of headache. Antifebrin, like most anilin derivatives, is a drug which should be employed with especial caution. It is official under the name of acetanilid, and its potency is sufficiently indicated by the fact that the maximum dose assigned to it is only three grains. There have been many cases of poisoning from the injudicious administration of this remedy, the symptoms produced by it being of the anilin type. The patient usually complains of giddiness, noises in the ears, throbbing in the temples, and a dull, heavy pain in the head. The face becomes livid, the lips are blue, and the pupils contracted. This is followed by symptoms of collapse, the face and extremities are cyanosed, the skin is covered with cold, clammy perspiration, the pulse is feeble, and respiration becomes shallow and frequent. There is no specific antidote, and after the administration of a brisk emetic the sufferer should be kept in a strictly recumbent position, and plied vigorously with stimulants. The effects are usually of considerable duration, and in one case the patient was not out of danger for fourteen hours. We are informed that there is a considerable demand for powders of this description, the purchasers being chiefly young women of a seamstress class. Many people acquire an unfortunate habit of dosing themselves with remedies of unknown composition, and this death under such sad circumstances may be taken as an indication that the custom is not one which can be indulged in with safety.

It is gratifying in heading a paragraph like the one that precedes this, to note that we have in ammonol a coal-tar product having unequaled antipyretic and analgesic properties. Also that in addition to these properties, ammonol is not only not depressive but is positively stimulating.—*Public Health Journal.*

